# An Electronic Screening and Brief Intervention for Hazardous and Harmful Drinking Among Swedish University Students: Reanalysis of Findings From a Randomized Controlled Trial Using a Bayesian Framework

**DOI:** 10.2196/14420

**Published:** 2019-12-17

**Authors:** Marcus Bendtsen

**Affiliations:** 1 Department of Medical and Health Sciences Linköping University Linköping Sweden

**Keywords:** Bayesian analysis, telemedicine, digital health, internet interventions, alcohol, randomized controlled trial

## Abstract

**Background:**

Due to a resurgent debate on the misuse of *P* values, the *Journal of Medical Internet Research* is hosting a standing theme issue inviting the reanalysis of (primarily digital health) trial data using a Bayesian framework. This first paper in this series focuses on an electronic screening and brief intervention (eSBI), targeting harmful and hazardous alcohol consumption, which student health care centers across Sweden have routinely administerd to all students during the past decade. The second *Alcohol Email Assessment and Feedback Study Dismantling Effectiveness for University Students* (AMADEUS-2) trial aimed to assess the effect of the eSBI on alcohol consumption among students who were harmful and hazardous drinkers. A two-arm randomized controlled trial design was employed, randomizing eligible participants to either a waiting list or direct access to an eSBI. Follow-up assessments were conducted 2 months after randomization. Subsequent analysis of the trial followed the conventional null hypothesis approach, and no statistical significance was found between groups at follow-up with respect to the number of standard drinks consumed weekly. However, in an unspecified sensitivity analysis, it was discovered that removing three potential outliers made the difference between the groups significant.

**Objective:**

The objective of this study is to reperform the primary and sensitivity analysis of the AMADEUS-2 trial using a Bayesian framework and to compare the results with those of the original analysis.

**Methods:**

The same regression models used in the original analysis were employed in this reanalysis (negative binomial regression). Model parameters were given uniform priors. Markov chain Monte Carlo was used for Bayesian inference, and posterior probabilities were calculated for prespecified thresholds of interest.

**Results:**

Null hypothesis tests did not identify a statistically significant difference between the intervention and control groups, potentially due to a few extreme data points. The Bayesian analysis indicated a 93.6% probability that there was a difference in grams of alcohol consumed at follow-up between the intervention and control groups and a 71.5% probability that the incidence rate ratio was <0.96. Posterior probabilities increased when excluding three potential outliers, yet such post hoc analyses were not necessary to show the preference toward offering an eSBI to harmful and hazardous drinkers among university students.

**Conclusions:**

The null hypothesis framework relies on point estimates of parameters. *P* values can therefore swing heavily, depending on a single or few data points alone, casting doubt on the value of the analysis. Bayesian analysis results in a distribution over parameter values and is therefore less sensitive to outliers and extreme values. Results from analyses of trials of interventions where small-to-modest effect sizes are expected can be more robust in a Bayesian framework, making this a potentially better approach for analyzing digital health research data.

**Trial Registration:**

International Standard Randomized Controlled Trial Number (ISRCTN) 02335307; http://www.isrctn.com/ISRCTN02335307

## Introduction

### Background

During the past decade, student health care centers across Sweden have routinely invited all students they serve to complete an electronic screening and brief intervention (eSBI) targeting harmful and hazardous alcohol consumption. Students are, on a yearly basis, invited via email to complete a 10-item questionnaire, after which they are given personal feedback alongside some advice on behavior change. The evidence for eSBIs generally indicates that they may have a small, yet positive effect on the amount of alcohol consumed in the short term (Cohen d=−0.17, 95% CI −0.27 to −0.18 [1]; Cohen d=−0.14, 95% CI −0.24 to −0.03 [2]) and the weighted mean difference of alcohol in grams (−16.59, 95% CI −23.70 to −9.48 [3]).

In 2011, the first *Alcohol Email Assessment and Feedback Study Dismantling Effectiveness for University Students* (AMADEUS-1) trial aimed to investigate the effect of this routine practice. An unconventional study design was used to target both treatment and non–treatment-seeking individuals as well as to mask trial participation and allow for baseline assessment effects to be measured. The trial, reported originally in 2013 [4-6], identified a small reduction in alcohol consumption and risky drinking among those who had been invited to assess their consumption compared to a no-contact control. A Bayesian reanalysis of the AMADEUS-1 trial has also been reported [7].

The unconventional trial design employed in the AMADEUS-1 trial necessitated inclusion of many individuals at follow-up who had decided not to complete the baseline assessment, as well as of nonharmful drinkers and abstainers. This prompted the AMADEUS-2 trial [8,9], which aimed to assess the effect of an eSBI on harmful and hazardous drinking among students.

### AMADEUS-2

The AMADEUS-2 trial [8,9] followed a more conventional two-arm randomized controlled trial design than did its predecessor AMADEUS-1. In March 2013, students in semesters 2, 4, and 6 at 9 colleges and universities in Sweden were sent an email (n=54,507) with an invitation to answer a single screening question regarding their alcohol consumption. The third item of the Alcohol Use Disorders Identification Test [10], which asks about the frequency of heavy episodic drinking, was used to screen participants for inclusion. Students were eligible if they had consumed at least four (female) or five (male) standard drinks twice a month or more often on a single occasion in the past 3 months. One standard drink is in Sweden defined as 12 grams of alcohol.

Eligible students who gave consent to take part in the trial were randomized into two groups: intervention and control. The intervention group was offered an eSBI immediately after randomization. They were asked to complete a 10-item questionnaire, which assessed their current consumption, after which they received feedback on their responses, including graphical representations of their current risk level, normative comparison with other students, and personal advice on how to reduce one’s consumption. The control group was told that they would receive the intervention in 2 months.

At follow-up, 2 months after the initial invitation, both groups were sent identical emails with an invitation to participate in the follow-up survey. The survey consisted of the same questionnaire and feedback that was offered to the intervention group at baseline.

### Concerns over the (Mis)use of *P* values

In 2017, Benjamin et al [11] (signed by 71 authors) recommended that the conventional threshold used for determining statistical significance should be lowered from .05 to .005. This recommendation was motivated by a growing concern that scientific findings are becoming less credible. Furthermore, the authors recommended that findings with *P* values between .05 and .005 should be considered suggestive evidence rather than being outright rejected.

This recommendation met critique, as others believed that trichotomization of evidence does not solve the issue of P-hacking, selective reporting, and publication bias [12,13]. These concerns resonate with the recent clarification from the American Statistical Association on the principles underlying *P* value reporting [14], Nuzzo’s summary in Nature [15], and a series of articles in the Journal of the American Statistical Association [16-20].

One approach that could potentially replace the *P* value dichotomization is the use of Bayesian inference, where evidence is considered as a continuous entity [21-25]. For this reason, the *Journal of Medical Internet Research* is inviting submissions to a special issue where authors are asked to reanalyze data from previous trials using a Bayesian framework and compare the analytical results with those of the original *P* value.

### Objective

The primary outcome in the AMADEUS-2 trial was self-reported weekly alcohol consumption at the 2-month follow-up. The main hypothesis was that the intervention group would report a lower weekly alcohol consumption than the control group at follow-up. An unplanned sensitivity analysis was also conducted, which excluded three data points considered outliers post hoc. The objective of this study is to redo the primary and sensitivity analysis using a Bayesian framework and contrast the results with those of the original analysis.

## Methods

### Bayesian model

In the original analysis of the AMADEUS-2 trial, negative binomial regression was used to contrast grams of alcohol consumed per week between the intervention and control groups. The primary model was adjusted for baseline variables. The same model was used in the enclosed Bayesian analysis, with uniform priors for all model parameters. Negative binomial regression with uniform priors used to contrast grams of alcohol per week is expressed by Equation 1:

g/week ~ NB(r,p)
log(r)=θ_0_ + θ_1_GROUP + θ_2_SEX + θ_3_AGE + θ_4_UNIVERSITY + θ_5_HED
θ_[0-5]_~uniform(–∞, +∞)
p~uniform(0, +∞)

Equation 1 presents the full specification of the model, where HED represents the number of heavy episodes of drinking per week at baseline, that is, the initial screening question.

The primary interest was the regression coefficient θ_1_ for the GROUP variable, that is, the expected difference in log count of grams of alcohol consumed between the intervention and control groups. By exponentiating this coefficient, we get the incidence rate ratio (IRR), which indicates by how much we should multiply the control group’s consumption to get the intervention group’s consumption. Thus, a value of exp (θ_1_) lower than 1 would suggest that the grams per week consumed for the intervention group was lower than that for the control group at the time of follow-up. Informed by the original analysis, thresholds for which the marginal posterior distribution of exp (θ_1_) should be inspected were chosen at 1, 0.96, and 0.92. The threshold of 1 was chosen to communicate whether offering the intervention was preferable to not doing so, and the thresholds 0.96 and 0.92 were chosen to indicate the magnitude of the difference between the two groups.

### Inference

Hamiltonian Monte Carlo, a type of Markov chain Monte Carlo (MCMC) technique, was used for Bayesian inference. The model was coded using Stan (Textbox 1) and run in R with RStan version 2.16.2. The data were one-hot encoded before being passed to Stan. No transformations were made to the variables.

When using MCMC for inference, we aim to draw samples from the posterior distribution of all model parameters. These samples can then be used to calculate how probable different values of these parameters are. For each model in the enclosed analysis, 50,000 iterations were run with 25,000 warmup iterations in four chains.

**Stan code used for inference for the parameters of the negative binomial model described in Equation 1.**

data {

int<lower=1> N; // Number of data items

int<lower=1> K; // Number of predictors

matrix[N,K] X;

int<lower=0> y[N]; // Response

}

parameters {

real<lower=0> phi; // Dispersion parameter

vector[K] beta;

}

model {

y ~ neg_binomial_2_log(X * beta, phi);

}

### Ethical Approval

This study was approved by the Regional Ethical Committee in Linköping, Sweden (No. 2013/46-31).

## Results

In total, 1605 eligible students agreed to take part in the trial, of which 825 were randomized to the intervention group and 780, to the control group. Two months after the initial invitation, 58% (931/1605) of trial participants completed the follow-up questionnaire.

### Original Analysis: Null Hypothesis Framework

Part of the original analysis of the AMADEUS-2 trial is presented in Table 1. Null hypothesis tests were two-tailed and assessed at the .05 threshold. No statistically significant difference was found between the intervention and control groups with respect to grams of alcohol consumed per week at follow-up (*P*=.13). To clarify, if we hypothesize that the population IRR is exactly 1, then the data collected in this trial are not extraordinary, that is, the probability of seeing these data is greater than 5%. According to convention, this does not allows us to reject the hypothesis that the IRR is exactly 1. The CI identifies a span of hypotheses that cannot be rejected, given the available data. Since the span includes both hypotheses of effect and no effect, the evidence is inconclusive.

**Figure 1:**
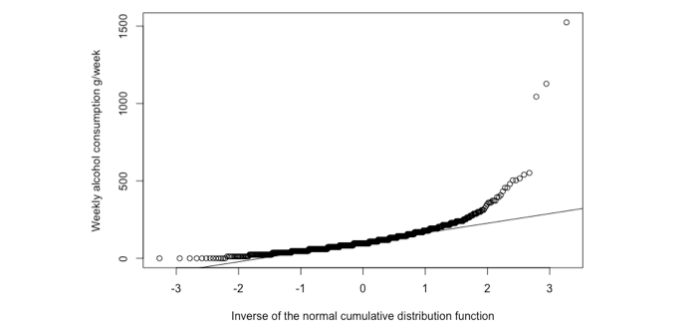
Unplanned sensitivity analysis identifying three potential outliers with respect to weekly alcohol consumption.

In an unplanned sensitivity analysis, data were graphically assessed for skewness (using Q-Q plots), and three potential outliers were identified (Figure 1): one in the intervention group (weekly consumption of 1044 g/week) and two in the control group (1128 g/week and 1524 g/week). These data suggest that the participants consumed over 80 standard drinks in a typical week. The difference between the groups was marginally statistically significant when these three outliers were excluded (*P* value=.049), with the intervention group on average reporting lower consumption than the control group.

**Table 1:** Original analysis of grams of alcohol consumer per week at follow-up compared between the intervention and control groups. When removing three potential outliers, the difference was marginally statistically significant.

	Intervention group (n=402), mean (SD)^a^	Control group (n=529), mean (SD)^a^	Incidence rate ratio^b^ (95% CI)	*P* value
Weekly alcohol consumption (g/wk)^b^	113.4 (81.1)	120.8 (86.4)	0.937 (0.861-1.019)	.13
Sensitivity analysis excluding three outliers	107.4 (73.4)	119.1 (81.3)	0.921 (0.848-1.000)	.049

^a^Mean and SD given by negative binomial regression.

^b^Incidence rate ratio given by negative binomial regression (adjusted for sex, age, university, and frequency of heavy episodic drinking at baseline).

### Bayesian Analysis

We recall from our discussion in the Methods section that Equation 1 represents the coefficient for the GROUP variable, that is, the difference between the intervention and control group in terms of log count of grams of alcohol consumed per week. We get the IRR by exponentiating this coefficient. The control group’s consumption is multiplied with the IRR to get the intervention groups consumption, thus an IRR less than 1 implies that the intervention group consumed less than the control.

**Figure 2:**
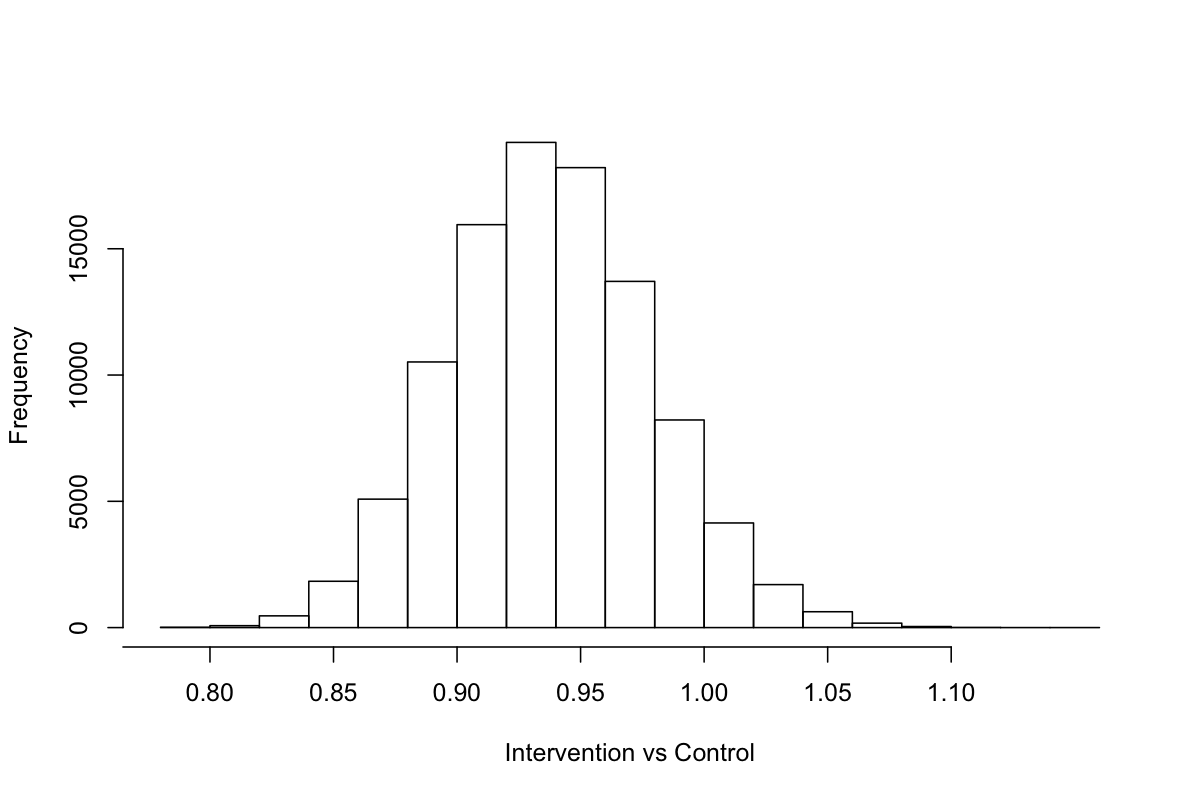
Samples from the posterior distribution of θ_1_ (exponentiated).

**Figure 3:**
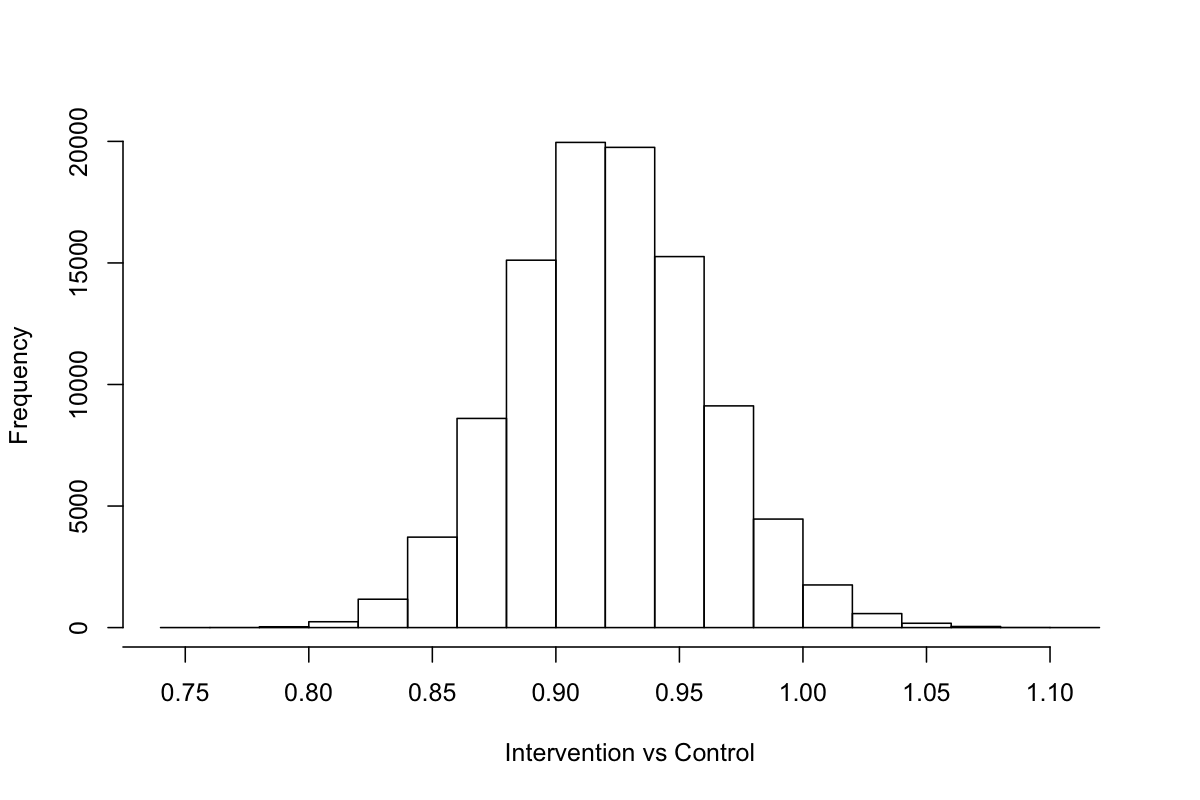
Samples from the posterior distribution of θ_1_ (excluding three potential outliers, exponentiated).

Histograms of the samples drawn from the posterior distribution of θ_1_ during MCMC are shown in Figure 2 (exponentiated) and samples drawn when excluding the three potential outliers are depicted in Figure 3 (exponentiated). These histograms should be interpreted as visualizing how plausible different values of θ_1_ are compared to one another. For instance, note how a strong majority of samples drawn were less than 1, indicating that it is more likely than not that the IRR is less than 1 when comparing the intervention and control groups. Thus, the model suggests that it is more likely than not, that the intervention group drank less than the control group.

For different IRR thresholds of interest, we can calculate the marginal posterior probability by simply counting the rate of samples that fall below or above a given threshold. In Table 2, we have calculated the marginal posterior probabilities for each of the predefined thresholds for the IRR. For instance, 17,875 samples were drawn below 0.96 when including the potential outliers (Figure 2), and we drew a total of 25,000 samples, resulting in a probability of 71.5% (17,875/25,000) that the IRR was less than 0.96.

No sampling issues during MCMC were found when inspecting trace plots (Multimedia Appendix 1).

**Table 2:** Bayesian analysis of incidence rate ratios comparing the intervention and control groups at follow-up.

	Intervention (n=402), mean (SD)	Control (n=529), mean (SD)	Probability^a^ (%)		
			Incidence rate ratio<1	Incidence rate ratio <0.96	Incidence rate ratio <0.92
Weekly alcohol consumption (g/wk)	113.4 (81.1)	120.8 (86.4)	93.6	71.5	33.9
Sensitivity analysis excluding three outliers	107.4 (73.4)	119.1 (81.3)	97.5	83.8	49.1

^a^Marginal posterior probabilities for incidence rate ratios comparing intervention and control groups, given by negative binomial regression (adjusted for sex, age university, and frequency of heavy episodic drinking at baseline, see Equation1).

## Discussion

### Null Hypothesis Testing

The original analysis of the AMADEUS-2 trial did not find a statistically significant difference between the intervention and control groups at follow-up (Table 2, *P* value=.13). A summary remark of the main analysis in the original publication was stated as follows [9]:

The study found no strong evidence of short-term effectiveness of the Swedish national system of proactive online alcohol intervention for university and college students. However, inspection of the confidence intervals for the primary outcome reveals that this study does not rule out an intervention effect of up to 13% reduction in total weekly alcohol consumption.

Thus, dichotomization leads us into a state of uncertainty: We cannot rule out that the intervention had no effect, yet we cannot conclude that the intervention had an effect.

The unplanned sensitivity analysis excluding outliers identified a marginally statistically significant difference; however, such unspecified analyses should be viewed with skepticism. It is generally impossible to know which data points should be considered correct, which are data entry errors, and which are malicious entries.

Although not included in the original analysis, we calculated the *P* value when excluding only the most extreme potential outlier (1524 g/week) and found that the difference between groups was then statistically significant with a *P* value of .04 (down from .13). The null hypothesis testing framework, and, in particular, *P* values, rely on point estimates of difference, that is, single values that are supposed to summarize the data. Such point estimates can be highly sensitive to single data points. Considering that policy decisions might be made based on this type of trial, we should feel uneasy knowing that statistical significance in a data set of 931 entries may rely on a single or a few study participants alone.

### Bayesian Analysis

The Bayesian analysis of the AMADEUS-2 trial (Figures 2 and 3, Table 2) suggests that that there is a 93.6% probability that the intervention group consumed less alcohol than the control group at follow-up in terms of the IRR. The data also suggest that the IRR was more likely than not to be less than 0.96. We may conclude that this difference is due to a positive effect of engaging in an eSBI, and this conclusion is licensed by the randomization component of the trial. However, the difference in point estimates of mean weekly alcohol consumption was approximately 7 grams between the intervention and control groups, suggesting that the eSBI had a lower mean effect than has been synthesized in meta-analyses [1-3].

When excluding the three entries with extreme levels of consumption, the probability of a difference increases. However, the difference is not extreme, partially because we are not relying on dichotomization, but mainly because in a Bayesian framework we look at the entire posterior distribution of parameters, rather than point estimates. The major benefit here is that we do not feel obligated to remove the potential outliers at all. Since analyses where outliers have been removed should be viewed with high skepticism, we can keep them in our data analysis while still obtaining similar results.

The posterior probabilities in Table 2 should be the basis for policy decision and be viewed in light of other factors, including alternative interventions and costs. The national system in Sweden used by the majority of student health care centers allows for eSBIs to reach tens of thousands of university students each year; however, the costs have been kept low by sharing a common platform. Given the high reach of the intervention and its low cost, there is a >90% probability of a positive effect in the trial, which may convince policy makers that the system should see continued use; however, this reasoning could not be established within the null hypothesis framework, since the evidence was found to be inconclusive.

### Limitations

The AMADEUS-2 trial was not sufficiently powered to obtain the prespecified effect size considered worth investigation. Approximately one quarter of the target sample size was recruited, creating a limitation on the possibility of detecting significant effect sizes. This also creates a limit for the Bayesian analysis, as the width of the posterior distribution, in general, decreases as the number of samples increase, allowing for narrower posterior distributions.

All analyses were performed under the intention-to-treat principle with complete cases, which assumes that data are missing at random. Although attrition analyses in the original publication did not find evidence against data missing at random, there was a difference in follow-up rates between the intervention group (404/825, 49.0%) and the control group (529/780, 67.8%), which should temper any strong conclusions from the original analysis and this reanalysis.

Finally, subjective measures were used to collect data at baseline and follow-up, which requires participants to recall their alcohol consumption in a typical week. Although such measurements may be subject to several sources of bias, such as recall and social desirability bias, it is the norm in brief interventions to use subjective measures, as in most cases, it is infeasible to collect biomarker data.

### Conclusions

The use of null hypothesis testing with *P* values has been the target for criticism for some time, not least due to the prevalent misinterpretation of *P* values and CIs [11,12,15-21]. Yet, the praxis stubbornly persists.

In the original publication of the AMADEUS-2 trial, it was acknowledged that it is challenging to reliably detect small effects and that the process may be subject to chance. Digital lifestyle interventions targeting large and sometimes non–treatment-seeking populations are generally expected to have a small-to-modest effect. Basing policy decisions on *P* values that may be highly sensitive to single data points may not be the most reliable way of deciding which evidence-based interventions should be recommended to the public.

## Appendix

Multimedia Appendix 1 Trace plots.

## Abbreviations

AMADEUS: Alcohol Email Assessment and Feedback Study Dismantling Effectiveness for University Students
eSBI: electronic screening and brief intervention
IRR: incidence rate ratio
MCMC: Markov chain Monte Carlo

## References

[1] Smedslund G, Wollscheid S, Fang L, Nilsen W, Steiro A, Larun L (2017). Effects of early, computerized brief interventions on risky alcohol use and risky cannabis use among young people. Campbell Systematic Reviews.

[2] Carey KB, Scott-Sheldon LAJ, Elliott JC, Garey L, Carey MP (2012). Face-to-face versus computer-delivered alcohol interventions for college drinkers: a meta-analytic review, 1998 to 2010. Clin Psychol Rev.

[3] Donoghue K, Patton R, Phillips T, Deluca P, Drummond C (2014). The effectiveness of electronic screening and brief intervention for reducing levels of alcohol consumption: a systematic review and meta-analysis. J Med Internet Res.

[4] McCambridge J, Bendtsen P, Bendtsen M, Nilsen P (2012). Alcohol email assessment and feedback study dismantling effectiveness for university students (AMADEUS-1): study protocol for a randomized controlled trial. Trials.

[5] Bendtsen P, McCambridge J, Bendtsen M, Karlsson N, Nilsen P (2012). Effectiveness of a proactive mail-based alcohol Internet intervention for university students: dismantling the assessment and feedback components in a randomized controlled trial. J Med Internet Res.

[6] McCambridge J, Bendtsen M, Karlsson N, White IR, Nilsen P, Bendtsen P (2013). Alcohol assessment and feedback by email for university students: main findings from a randomised controlled trial. Br J Psychiatry.

[7] Bendtsen M (2019). Electronic Screening for Alcohol Use and Brief Intervention by Email for University Students: Reanalysis of Findings From a Randomized Controlled Trial Using a Bayesian Framework. J Med Internet Res.

[8] McCambridge J, Bendtsen M, Karlsson N, White IR, Bendtsen P (2013). Alcohol assessment & feedback by e-mail for university student hazardous and harmful drinkers: study protocol for the AMADEUS-2 randomised controlled trial. BMC Public Health.

[9] Bendtsen P, Bendtsen M, Karlsson N, White IR, McCambridge J (2015). Online Alcohol Assessment and Feedback for Hazardous and Harmful Drinkers: Findings From the AMADEUS-2 Randomized Controlled Trial of Routine Practice in Swedish Universities. J Med Internet Res.

[10] Bush K (1998). The AUDIT Alcohol Consumption Questions (AUDIT-C): An Effective Brief Screening Test for Problem Drinking. Arch Intern Med.

[11] Benjamin DJ, Berger JO, Johannesson M, Nosek BA, Wagenmakers E, Berk R, Bollen KA, Brembs B, Brown L, Camerer C, Cesarini D, Chambers CD, Clyde M, Cook TD, De Boeck P, Dienes Z, Dreber A, Easwaran K, Efferson C, Fehr E, Fidler F, Field AP, Forster M, George EI, Gonzalez R, Goodman S, Green E, Green DP, Greenwald AG, Hadfield JD, Hedges LV, Held L, Hua Ho T, Hoijtink H, Hruschka DJ, Imai K, Imbens G, Ioannidis JPA, Jeon M, Jones JH, Kirchler M, Laibson D, List J, Little R, Lupia A, Machery E, Maxwell SE, McCarthy M, Moore DA, Morgan SL, Munafó M, Nakagawa S, Nyhan B, Parker TH, Pericchi L, Perugini M, Rouder J, Rousseau J, Savalei V, Schönbrodt FD, Sellke T, Sinclair B, Tingley D, Van Zandt T, Vazire S, Watts DJ, Winship C, Wolpert RL, Xie Y, Young C, Zinman J, Johnson VE (2017). Redefine statistical significance. Nat Hum Behav.

[12] Amrhein V, Greenland S (2017). Remove, rather than redefine, statistical significance. Nat Hum Behav.

[13] McShane B, Gal D, Gelman A, Robert C, Tackett J (2019). Cornell Univeristy - arXiv.org.

[14] Wasserstein RL, Lazar NA (2016). The ASA's Statement on -Values: Context, Process, and Purpose. The American Statistician.

[15] Nuzzo R (2014). Scientific method: Statistical errors. Nature.

[16] Berry D (2017). A P-Value to Die For. J Am Stat Assoc.

[17] Briggs WM (2017). The Substitute for P-Values. J Am Stat Assoc.

[18] Gelman A, Carlin J (2017). Some Natural Solutions to the P-Value Communication Problem - and Why They Won’t Work. J Am Stat Assoc.

[19] Laber EB, Shedden K (2017). Statistical Significance and the Dichotomization of Evidence: The Relevance of the for Statisticians. J Am Stat Assoc.

[20] McShane BB, Gal D (2017). Rejoinder: Statistical Significance and the Dichotomization of Evidence. J Am Stat Assoc.

[21] Bendtsen M (2018). A Gentle Introduction to the Comparison Between Null Hypothesis Testing and Bayesian Analysis: Reanalysis of Two Randomized Controlled Trials. J Med Internet Res.

[22] Spiegelhalter D, Abrams K, Myles J (2003). Bayesian Approaches to Clinical Trials and Health-Care Evaluation.

[23] Kruschke JK (2013). Bayesian estimation supersedes the t test. J Exp Psychol Gen.

[24] Browne EN, Rathinam SR, Kanakath A, Thundikandy R, Babu M, Lietman TM, Acharya NR (2017). A Bayesian Analysis of a Randomized Clinical Trial Comparing Antimetabolite Therapies for Non-Infectious Uveitis. Ophthalmic Epidemiol.

[25] Goodman SN, Sladky JT (2005). A Bayesian approach to randomized controlled trials in children utilizing information from adults: the case of Guillain-Barré syndrome. Clin Trials.

