# Supplementary material for: An Electronic Screening and Brief Intervention for Hazardous and Harmful Drinking Among Swedish University Students: Reanalysis of Findings From a Randomized Controlled Trial Using a Bayesian Framework
Source: J Med Internet Res. 2019 Dec 17;21(12):e14420. doi: 10.2196/14420 (PMC6938590; doi:10.2196/14420)
Supplement: Multimedia Appendix 1 [file jmir_v21i12e14420_app1.docx]

# Appendix a – Trace plots

Trace plots can be useful to diagnose issues in sampling when using Markov chain Monte Carlo methods. Figure 3 and Figure 4 show trace plots for the two included analyses. Specifically, since there are no visible trends in the plots, the sampling was likely well dispersed over parameter values.


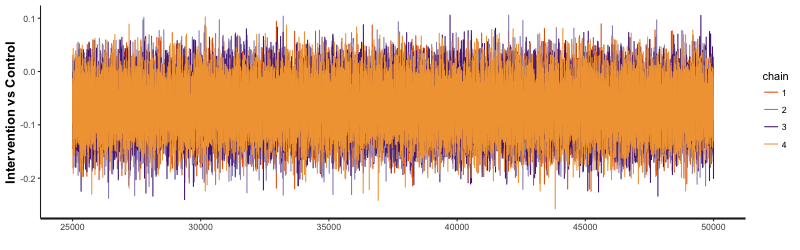


**Figure 4** - Trace plot of samples drawn during Markov chain Monte Carlo inference (including potential outliers).


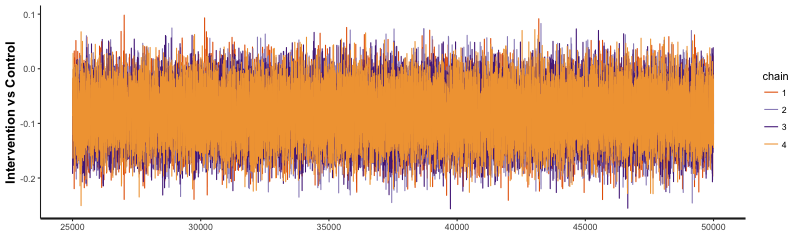


**Figure 5** - Trace plot of samples drawn during Markov chain Monte Carlo inference (excluding potential outliers).
